# Effectiveness of photochemical and sonochemical processes in degradation of Basic Violet 16 (BV16) dye from aqueous solutions

**DOI:** 10.1186/1735-2746-9-14

**Published:** 2012-11-29

**Authors:** Zahra Rahmani, Majid Kermani, Mitra Gholami, Ahmad Jonidi Jafari, Niyaz Mohammad Mahmoodi

**Affiliations:** 1 Department of Environmental Health Engineering, School of Public Health, Tehran University of Medical Sciences, Tehran, Iran; 2 Department of Environmental Health Engineering, School of Public Health, Center for Water Quality Research (CWQR), Institute for Environmental Research (IER), Tehran University of Medical Sciences, Tehran, Iran; 3 Department of Environmental Health Engineering, School of Medical Sciences, Tarbiat Modares Univesity, Tehran, Iran; 4 Department of Environmental Research, Institute for Color Science and Technology, Tehran, Iran

**Keywords:** Basic violet 16, Decolorization, Hydroxyl radical, US/H_2_O_2_, UV/H_2_O_2_

## Abstract

In this study, degradation of Basic Violet 16 (BV16) by ultraviolet radiation (UV), ultrasonic irradiation (US), UV/H_2_O_2_ and US/H_2_O_2_ processes was investigated in a laboratory-scale batch photoreactor equipped with a 55W immersed-type low-pressure mercury vapor lamp and a sonoreactor with high frequency (130kHz) plate type transducer at 100W of acoustic power. The effects of initial dye concentration, concentration of H_2_O_2_ and solution pH and presence of Na_2_SO_4_ was studied on the sonochemical and photochemical destruction of BV16 in aqueous phase. The results indicated that in the UV/H_2_O_2_ and US/H_2_O_2_ systems, a sufficient amount of H_2_O_2_ was necessary, but a very high H_2_O_2_ concentration would inhibit the reaction rate. The optimum H_2_O_2_ concentration was achieved in the range of 17 mmol/L at dye concentration of 30 mg/L. A degradation of 99% was obtained with UV/H_2_O_2_ within 8 minutes while decolorization efficiency by using UV (23%), US (<6%) and US/H_2_O_2_(<15%) processes were negligible for this kind of dye. Pseudo-first order kinetics with respect to dyestuffs concentrations was found to fit all the experimental data.

## Introduction

Due to incomplete fixation of dyes on fibers during the coloring and washing steps, it is estimated that 1-15% of dyestuffs used in textile industries end up in wastewaters [1]. Dye pollutants are generally resistant to biological degradation and cannot be thoroughly processed in wastewater treatment plants [2]. Ultimate accumulation of these molecules in human, through marine life, may pose long-term problems [3-5]). There are many different classes of dyes, such as azo, Reactive, metal complex, azo metal complex and cationic and anionic dyes [6]. Basic Violet 16 (BV 16) is a cationic dye, highly water soluble and nonvolatile. It is widely used in textile, leather industries, in preparing carbon paper, bull pen, stamp pad inks and paints, and is also a well-known water tracer fluorescent [7]. BV 16 directly harms skin, eyes, and gastrointestinal respiratory tract. It also provokes phototoxic and photo allergic reactions [8]. Its carcinogenicity, reproductive and developmental toxicity, neurotoxicity and chronic toxicity towards humans and animals have also been experimentally proven [9].

Many physical and chemical methods, such as adsorption, coagulation, floatation, solvent extraction and hyperfiltration have been applied to remove dyes [10,11]. However, these methods merely transfer the pollutant from one media to another. Alternate technologies popularly known as Advanced Oxidation Processes (AOPs) have been extensively explored for complete demineralization of these compounds for the past few decades. AOPs are characterized by production of OH (hydroxyl) radical as primary oxidant. The OH radicals are extremely reactive species and powerful oxidizing agent [12-14].

Application of Ultraviolet irradiation (UV), as an AOP, to the decolorization of organic compounds is intrinsically interesting: most organic compounds might transform, and even undergo complete destruction in the presence of UV irradiation [15,16]. Another interesting aspect of photolysis is the possibility of application of photocatalysts, such as TiO2, which greatly enhances the rate of oxidation of dyes by UV [17-19].

Sonication is a relatively novel AOP based on applying low to medium frequency (typically in the range 20–1000 kHz) and high energy ultrasound to catalyze the destruction of organic pollutants in waters [20]. Sonochemical treatment typically operates at ambient conditions and does not require the addition of extra chemicals or catalysts [21,22]. Acoustic cavitation, that is, formation, growth, and collapse of bubbles in liquid is the basis of sonication. There are several theories explaining the mechanisms of sonication induced chemical reactions. One popular theory, the hot spot approach, states that each bubble acts as a localized micro reactor with three regions: (1) a hot gaseous nucleus with temperatures up to several thousand degrees and pressures in excess of one thousand atmospheres, (2) an interfacial region, and (3) the bulk solution [23]. Two main reaction mechanisms are proposed: (i) oxidation by OH radicals at all reaction sites, and (ii) thermolysis inside cavitation bubbles and at the bubble surface.

To the best of our knowledge, no study has been published on the decolorization of BV16 using high frequency (130 kHz, 100 W) sonochemical processes and UV radiation (low-pressure, 55W) either alone or in conjunction with hydrogen peroxide. Therefore, the main objective of this study was to evaluate the application of high frequency ultrasound and low pressure mercury vapor lamp for the degradation of BV16 either alone or in conjunction with hydrogen peroxide in a pilot scale reactor. The effects of operating parameters such as pH, initial dye concentration, hydrogen peroxide dosage and salt (Na_2_SO_4_) on the decolorization were also studied. The optimal conditions for each process including the time required for complete degradation were determined. Furthermore, comparisons were made between applied processes in terms of degradation efficiency.

## Materials and methods

**Figure 1:**
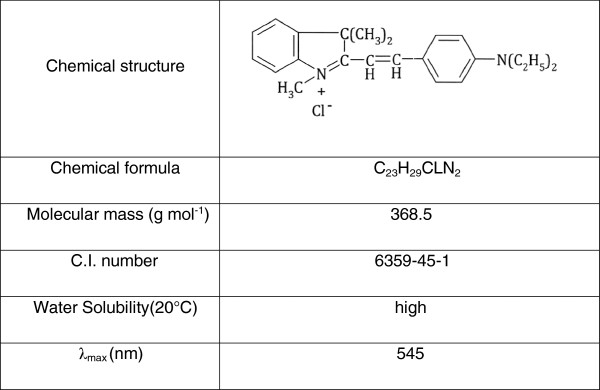
Physical and chemical properties of BV16.

Commercial C.I. Basic Violet 16 (BV16) was obtained from Alvan Sabet Co. (Hamedan, Iran) and used without further purification. The most important physical and chemical properties of this cationic dye are presented in Figure 1. All other chemicals used in the experiments were obtained from Merck Chemical Co, Germany. Dye solutions were prepared using deionized water with the initial concentration of 10–100 mg/ L. The pH of the solutions was checked using a pH meter (HQ40d, USA) and adjusted by adding concentrated H_2_SO_4_ where needed. Sonication was achieved at a frequency of 130 kHz (100W) with an ultrasonic generator (Elma CD-4820, Germany) with a piezoelectric transducer having a diameter of 5cm fixed at the bottom of the vessel.

**Figure 2:**
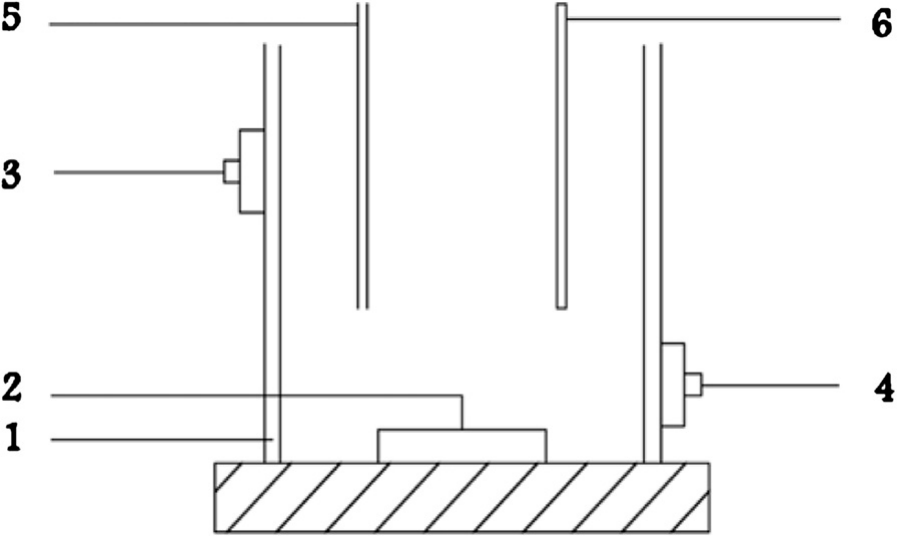
Schematic diagram of sonoreactor.

The photo- degradation studies were carried out in a semi- batch reactor system. The photoreactor was made from stainless steel, which was available for the high reflection of the radiation (Figure 2).

The reactor chamber was also cylindrical with a capacity of 2.5 L and a length of 86 cm, with an inner and outer diameter of 7.6 and 7.8 cm, respectively. Irradiation was achieved by a low-pressure mercury lamp (Philips, TUV 55W HQ) located axially in the center of reactor and immersed in the solution. The light length of the lamp and quartz sleeve diameter of 30 mm was same with reactor that emits approximately 90% of its radiation at 253.7 nm. The reaction chamber was filled with the mixture, which was placed between the reactor walls and UV lamp system.

The reactors were filled with constant concentration of 30 mg/ L cationic dye (BV16) to simulate a dye containing industrial wastewater. For runs using UV/H_2_O_2_ and US/H_2_O_2_, hydrogen peroxide at different amounts was injected in the feed tanks. The initial H_2_O_2_ concentration varied in the range of 1–50 mmol/L. The reaction was performed at pH 3 and 4.5 to test for its effects on the reaction rate. Also, for studding of the salt addition effects on the degradation of dye with various salt (Na_2_SO_4_) concentrations in the range of 0.5-2 g/ L was used.

To measure the decolorization of dye solution, samples were withdrawn from the reactors at certain time intervals (till 120 min) and analyzed immediately. Decolorization of solution was measured with a UV/VIS spectrophotometer (CECIL EC 7400, England) via the decrease in maximum absorbance of the dye (545nm at pH 3 and 4.5). The reactor was maintained at room temperature. For considering of the mineralization of basic violet 16 (BV16), COD (chemical oxygen demand) test was used. The COD determination was carried out according to standard methods [24].

## Results

### Initial H_2_O_2_ concentration effects

**Figure 3:**
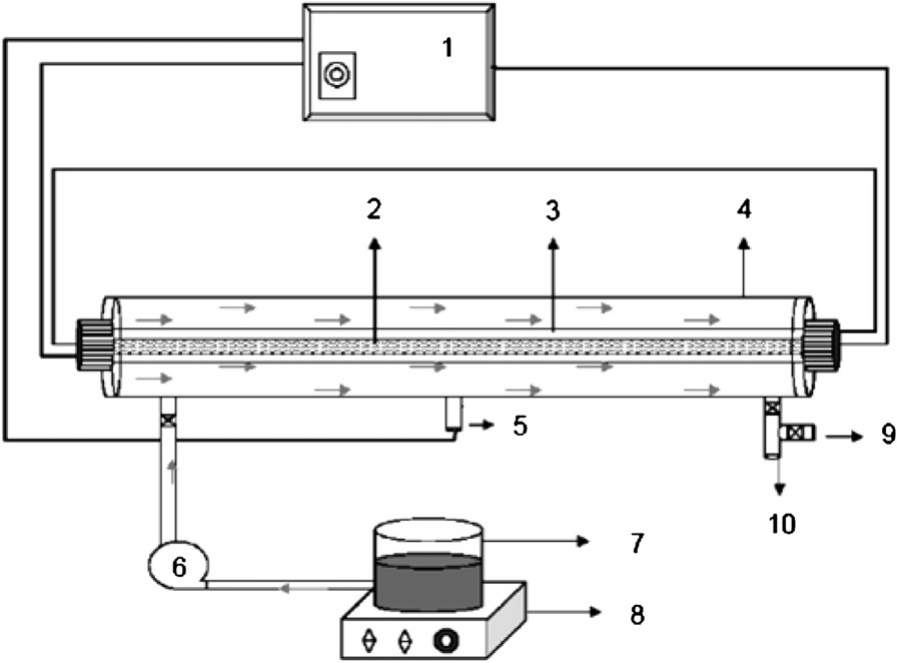
BV16 degradation as a function of hydrogen peroxide by US and US/H_2_O_2_ processes, dye concentration=30 mg/ L and pH=4.5.

Results with UV/H_2_O_2_ and US/H_2_O_2_ processes indicate that the oxidation was exclusively due to hydroxyl radical attack when hydrogen peroxide was in excess. BV16 degradation rate by US and US/H_2_O_2_ was strongly dependent on initial concentration of hydrogen peroxide, as shown in Figure 3.

**Figure 4:**
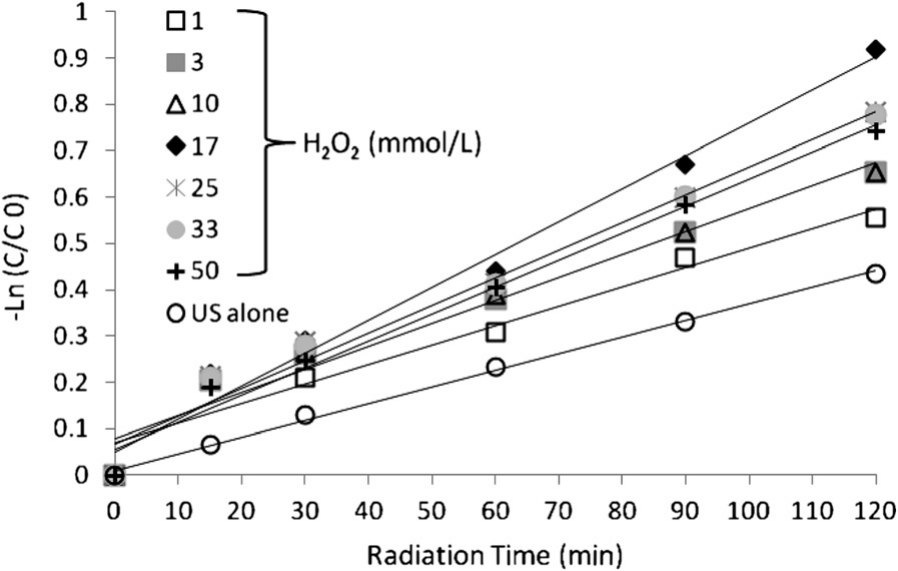
BV16 degradation as a function of hydrogen peroxide by UV and UV/H_2_O_2_ processes, dye concentration=30 mg/L and pH=4.5.

Figure 4, Shows UV/H_2_O_2_ is more efficient than UV light alone for BV16 decolorization.

**Figure 5:**
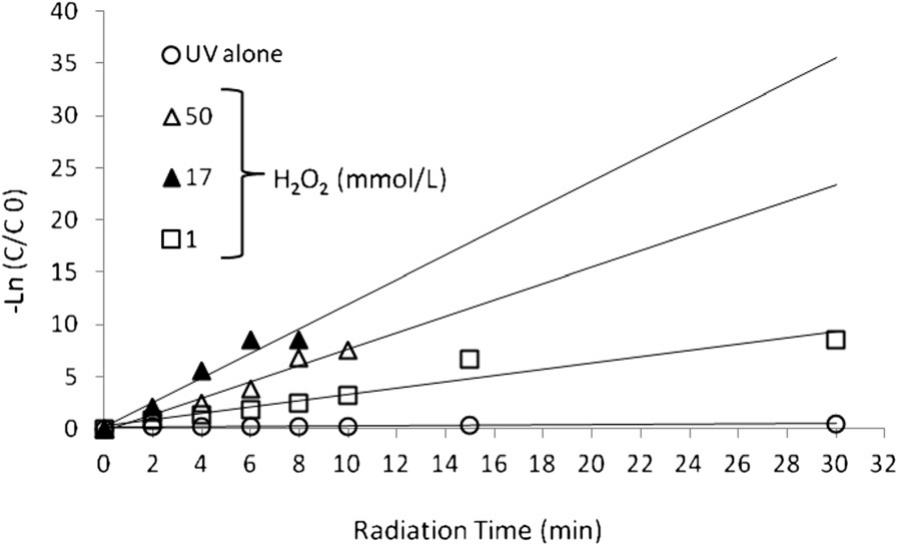
Comparison of US and UV for dye removal at different time, dye concentration=30 mg/ L and pH=4.5.

Comparison of photo-degradation and sono-degradation processes efficiencies showed that photodegradation of BV16 was more efficient than sono-degradation (Figure 5).

### Effect of solution pH

**Figure 6:**
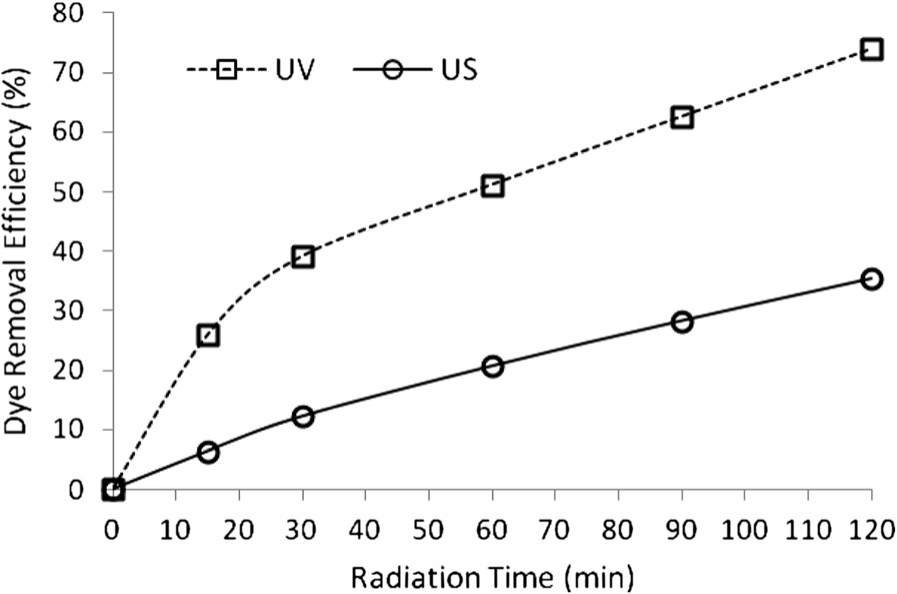
BV16 degradation as a function of pH by UV/H_2_O_2_ and US/H_2_O_2_ processes, dye concentration=30 mg/ L and H_2_O_2_ Concentration=17 mmol/ L.

Dye decolorization was carried out at pH 3 and 4.5 during oxidation processes (Figure 6). It is notable that in US/H_2_O_2_ process, decolorization depends strongly on the solution pH and is substantially reinforced at acidic conditions. It can be seen that UV/H_2_O_2_ process was more efficient than US/H2O2 process. Results showed that in UV/H_2_O_2_ process, natural pH (4.5) was the optimum pH, because the degradation of BV16 in pH = 3 was equal to natural pH.

### Effect of initial dye concentration

**Figure 7:**
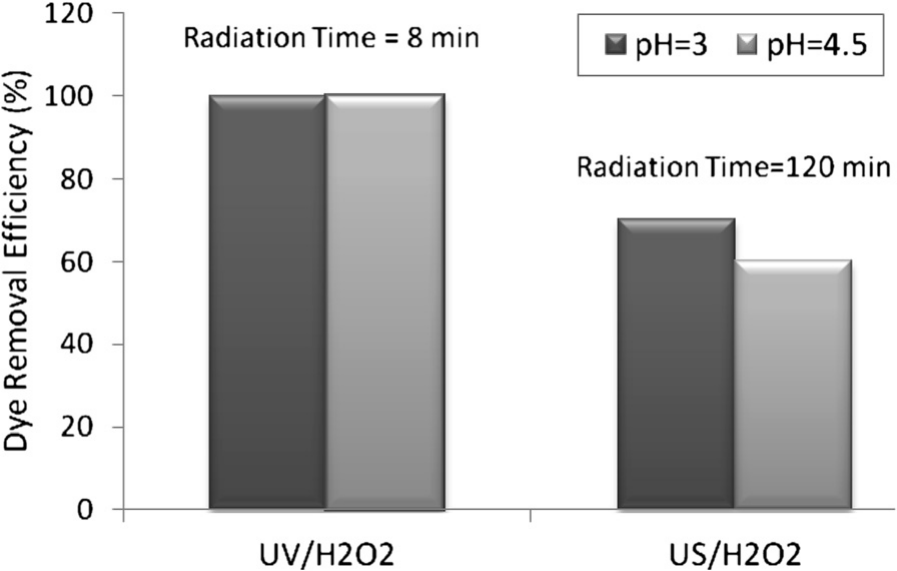
**Effect of initial dye concentrations on UV/H_2_O_2_ process efficiency at different radiation times.** H_2_O_2_ concentration=17 mmol/L and pH=4.5.

**Figure 8:**
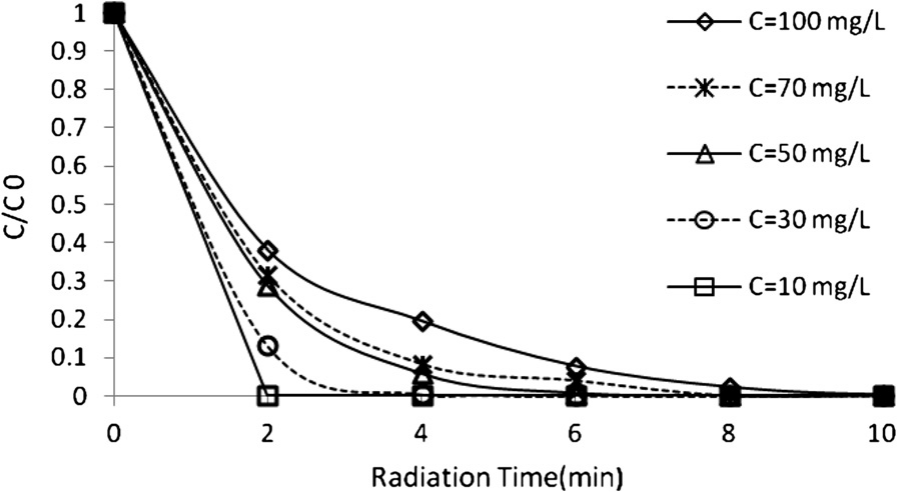
**Effect of initial dye concentrations on US/H_2_O_2_ process efficiency at different radiation times.** H_2_O_2_ concentration=17 mmol/ L and pH=3.

To study the effect of dye concentration on the rate of decolorization, the dye concentration (C_0_) was varied between 10 mg/ L and 100 mg/ L, while the other variables were kept constant (Figures 7 and 8).

### Comparison of reaction rate

**Figure 9:**
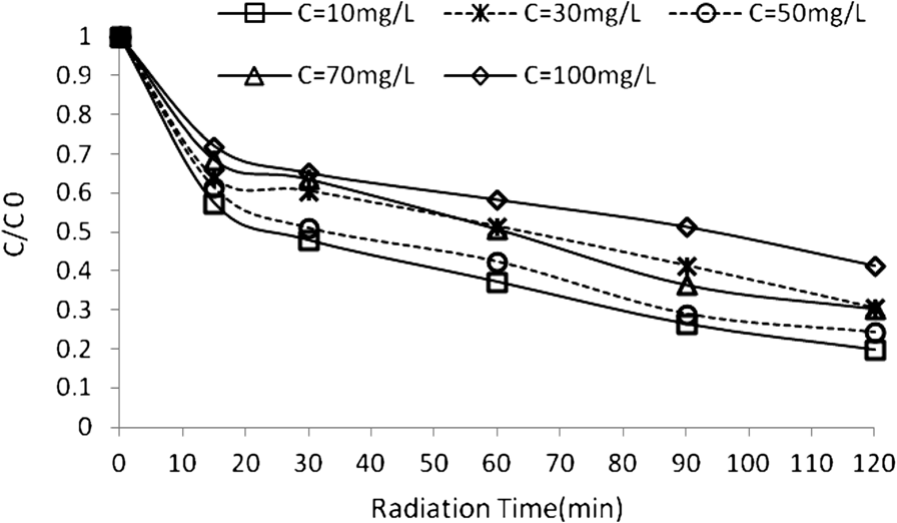
Pseudo first order plot for degradation of dye by different processes, dye concentration=30 mg/ L, H_2_O_2_ Concentration=17 mmol/ L and pH=4.5.

The value of pseudo first order kinetic constant was obtained by fitting the experimental data at optimum condition of systems in Figure 4, 5 and 6 to a straight line in Figure 9. These results are summarized in Table 1.

**Table 1:** Pseudo first order kinetic and half-life for the dyes degradation processes

**Type of process**	**K (min**^**-1**^**)**	***T***_**1/2**_**(min)**	**R**^**2**^
US	0.0036	>120	0.9978
US/H_2_O_2_	0.0071	90	0.9834
UV	0.0098	60	0.9818
UV/H2O2	1.4534	<2	0.99

**Figure 10:**
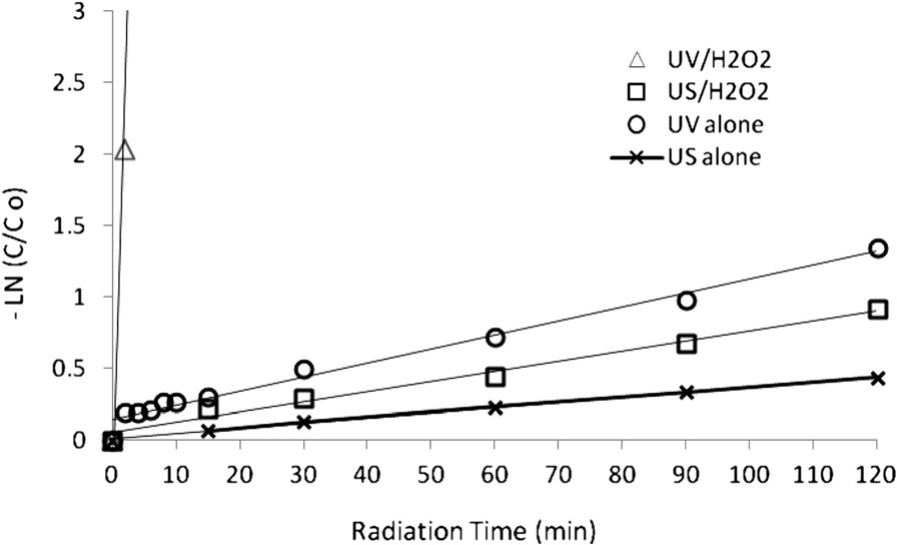
The color removal by US/H_2_O_2_ process in the presence of sodium sulfate (Na_2_SO_4_), dye concentration=30 mg/ L, H_2_O_2_ concentration=17 mmol/L and pH=3.

Figure 10 shows the effect of Na_2_SO_4_ addition on the decolorization kinetics of dye by US/H_2_O_2_.

### Effect of sodium sulphate addition

**Figure 11:**
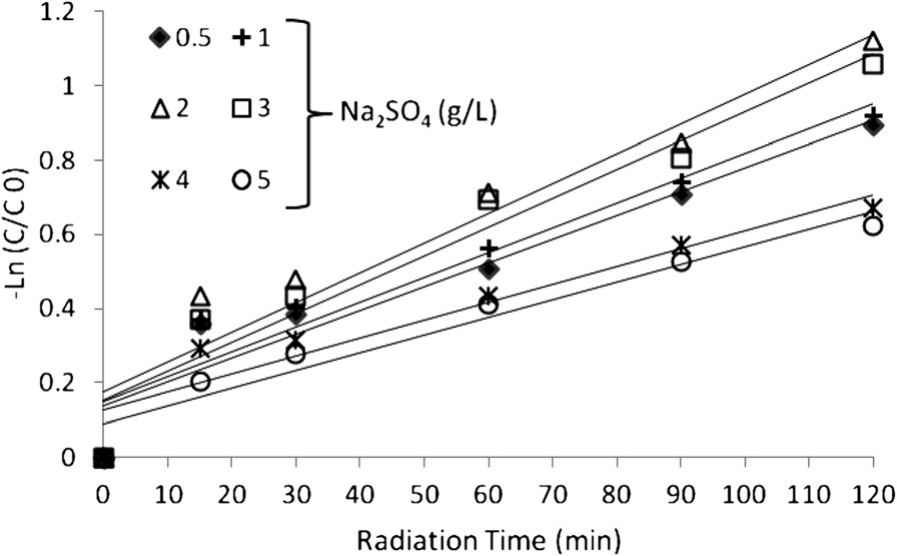
Comparison of US/H_2_O_2_ and UV/H_2_O_2_ processes for COD removal at different radiation time, H_2_O_2_ Concentration=17 mmol/ L, dye concentration=30 mg/ L and Na_2_So_4_ = 2 g/ L.

As it can be seen in Figure 11, addition of Na_2_SO_4_ slightly enhanced the degradation of BV16 up to 2 g/ L when compared with the absence of Na_2_SO_4_ salt.

### Dye degradation and mineralization

**Figure 12:**
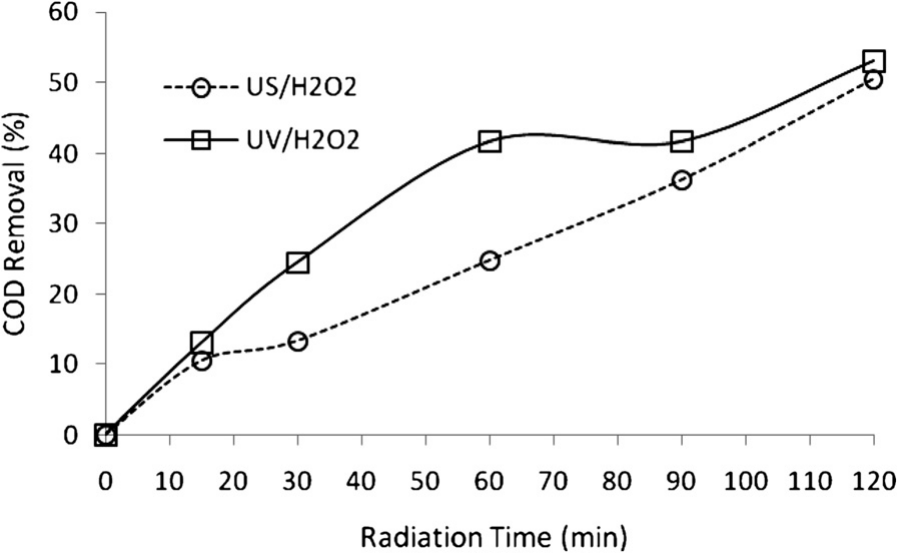
Comparison of US/H_2_O_2_**and UV/H_2_O_2_processes for COD removal at different time (H_2_O_2_Concentration=17 mmol L^-1^, Dye concentration=30 mg L^-1^**, Na_2_So_4_**= 2 g L^-1^**).

The rate of COD removal of BV16 by UV/H_2_O_2_ and US/H_2_O_2_ processes is shown in Figure 12.

## Discussion

The combination of UV with H_2_O_2_, which leads to photolysis of H_2_O_2_ and rapid production of hydroxyl radicals [25], is necessary to perform dye decolorization, even for the higher dye concentrations. These results are in good agreement with other findings in literature, such as [26].

This is encountered during the destruction of not only dyes but also many organic compounds as well [27]. The minimal effect of US alone on the decolorization may be attributed to the fact that ultrasound waves hinder the development of hydroxyl radicals, [28].

BV16, like other highly soluble compounds, reactions inside or in the vicinity of the bubble (where fast thermal decomposition and increased concentrations of radicals exist) are unlikely to occur to an appreciable extent and therefore its degradation will be driven by hydroxyl radical-mediated secondary activity in the liquid bulk [29].

Further addition of H_2_O_2_ leads to a decrease in reaction rate in both UV/H_2_O_2_ and US/H_2_O_2_ experiments. In our experiments, the optimum concentration of H_2_O_2_ was found to be 17 mM for both processes. This trend can be explained by the fact that H_2_O_2_ itself acts as an effective hydroxyl radical scavenger. At some H_2_O_2_ concentration, the competition between scavenging of radicals by H_2_O_2_ and degradation of dye reaches a point with optimal dye degradation. This optimal concentration of H_2_O_2_ depends on chemical structure (reactivity and photosensitivity) of the dye. Photodegradation of BV16 was more efficient than the sonodegradation (Figure 4). UV involves the participation of a more diverse range of reactive species (i.e., radicals and electrons) than the US [16,30].

In further experiments, decolorization was studied at different pHs (3 and 4.5) (Figure 5). It is notable that in US/H_2_O_2_ process, decolorization depends strongly on the solution pH and is substantially reinforced at lower pH (pH=3), while hindered at higher pH (pH=4.5). This acceleration is probably associated the hydrophobic character of the resulting molecule enhances its reactivity under oxidation processes [31]. Moreover, under acidic pH, hydroxyl radical is the predominant reactive oxidant [32]. In contrast to US/H_2_O_2_ observations, results showed that in UV/H_2_O_2_ process, natural pH (4.5) was the optimum pH.

In UV/H_2_O_2_, the rate of degradation decreases when initial dye concentration increases. This is attributed to the absorbtion of UV by dye and prevention of radical formation [33]. In US/H_2_O_2_, the degradation rate depends on the initial concentration of dye.

As expected, by increasing the dye concentration, the dye degradation rate increases to an optimum value and then decreases. The presumed reason is that when the initial concentration of dye is increased, the competition between the dye intermediates and parent dye for hydroxyl radical becomes intense owing to the non-selective reactivity of hydroxyl radical. Additionally, in the US process, with increasing initial dye concentration the cavities approaches saturation [34] and [4].

Thus, the time for complete decolorization would be longer for higher initial dye concentrations [31]. At lower dye concentrations, a considerable part of these OH radicals will recombine yielding H_2_O_2_ and the degradation is carried out in the bulk of the solution where there is a lower concentration of OH radicals because only about 10% of the OH radicals generated in the bubble can diffuse into the bulk solution which conduct to lower degradation rates [12,32,35].

The loss of dye was observed as a function of irradiation time and data were fitted to a pseudo first order rate model according to following equation [36,37].

(1) $$\text{In}\left(\frac{c}{c_{0}}\right)\;=-\text{k't}$$

The slope of the plot of $\text{In}\left(\frac{c}{c_{0}}\right)$ versus time gives the value of rate constant k', min^-1^ (As seen from figure k depends on the concentration of H_2_O_2_), so the reaction follows a pseudo-first order scheme. Here, C_o_ denotes the initial concentration in milligrams per liter, and C is the concentration value in milligrams per liter at time t. In general, as can be seen in Figure 8, UV/H_2_O_2_ system needs very low irradiation time to complete decolorization relative to UV, US/H_2_O_2_ and US systems, the UV/H_2_O_2_ process is more economical than the other studied systems from energy consumption point of view.

These results are in good agreement with other findings in the literatures [22,29,38,39]. To assess the effect of dissolved inorganic anions on the decolorization of dyes, sodium sulfate (Na_2_SO_4_) was used.

The results indicated that in the UV/H_2_O_2__process_, the addition of Na_2_SO_4_ does not affect decolorization kinetics. However, in sonication experiment, Na_2_SO_4_ altered the rate of the degradation of dye. As it can be seen in Figure 9, addition of up to 2 g/ L Na_2_SO_4_ slightly enhanced the degradation of BV16.

The positive effect of Na_2_SO_4_ on the destruction of BV16 slightly decreased in the concentration range 2–5 g/ L of the salt, however, the degradation rate remained higher than that obtained without Na_2_SO_4_. The presence of salt increases the hydrophilicity of aqueous phase and so push the dye to the bulk- bubble interface, increases the surface tension and ionic strength of the aqueous phase and decreases the vapour pressure [40,41].

All these factors help in collapsing of the bubbles more violently, resulting in higher degradation of dye. Excessive amount of Na_2_SO_4_ may interfere with the introduction of ultrasound into the liquid [42]. As depicted in the Figure 10, the UV/H_2_O_2_ outperforms US/H_2_O_2_ in terms of COD removal, which can be explained by the fact that the former process produces more hydroxyl radicals and, as a result, is more efficient for mineralization. the remaining COD under UV/H_2_O_2_ oxidation conditions (regiments) after 120 minutes (about 45% in 120 min) indicated that the COD removal could not be completed within 120 mins, indicating various organic intermediates were produced during the degradation of dye [31,43]. These results imply that the degradation intermediate products of BV16 are recalcitrant toward photochemical and sonochemical treatment [12].

## Conclusions

In this experimental study, decolorization of Basic violet 16 (BV16) was investigated by using several advanced oxidation processes: US, US plus H_2_O_2_, UV and UV plus H_2_O_2_.

Optimal operating condition for each process was established. The conclusions drawn from this study can be summarized as follows:

The decolorization efficiency proceeded very slowly when US, US plus H_2_O_2_ and UV processes were used.

UV plus H_2_O_2_ process was found to be a suitable treatment method for complete decolorization for BV16.

The decolorization efficiency was increased with increasing H_2_O_2_ concentration; however, the marginal benefit became decreasing with further increasing of H_2_O_2_ due to the scavenging effect of excess H_2_O_2_.

The rate of color decay followed pseudo-first order kinetics with respect to the UV-visible absorption of the test dye during reaction.

The results indicated that the addition of Na_2_SO_4_ does not show any positive or negative effect upon decolorization kinetics in the UV/H_2_O_2_ process. However, in the US/H_2_O_2_ process, addition of inorganic anion (Na_2_SO_4_), up to 2 g L−1, slightly enhanced the degradation of BV16, compared to the absence of Na_2_SO_4_ salt.

Though decolorization will achieve perfection within 15 minutes in UV/H_2_O_2_, COD will be only reduced to 53% in 120 mins. The partial reduction of COD represents the production of small organic molecular fragments and incomplete mineralization of dye by UV/H_2_O_2_ and US/H_2_O_2_ processes.

## Competing interests

The authors declare that they have no competing interests.

## Authors’ contributions

ZR, MK, MG , AJJ and NMM carried out the article with the title of: Effectiveness of photochemical and sonochemical processes in degradation of basic violet 16 (bv16) dye from aqueous solutions, participated in the sequence alignment and drafted the manuscript. All authors read and approved the final manuscript.
